# Characterisation of the molecular properties of scleroglucan as an alternative rigid rod molecule to xanthan gum for oropharyngeal dysphagia

**DOI:** 10.1016/j.foodhyd.2019.105446

**Published:** 2020-04

**Authors:** Xinxin Li, Yudong Lu, Gary G. Adams, Hanne Zobel, Simon Ballance, Bettina Wolf, Stephen E. Harding

**Affiliations:** a National Centre for Macromolecular Hydrodynamics, School of Biosciences, University of Nottingham, Sutton Bonington Campus, Loughborough, LE12 5RD, UK; b Division of Food, Nutrition and Dietectics, School of Biosciences, University of Nottingham, Sutton Bonington Campus, Loughborough, LE12 5RD, UK; c School of Health Sciences, University of Nottingham, Queen's Medical Centre, Nottingham, NG7 2HA, UK; d Nofima AS, Norwegian Institute of Food, Fisheries and Aquaculture Research, Ås, Norway; e Kulturhistorisk Museum, Universitetet i Oslo, Postboks 6762, St. Olavs Plass, 0130, Oslo, Norway

## Abstract

Scleroglucan, a neutral β(1–3) glucan with β(1–6) glucan branches every third residue, is being considered as an alternative rod-like, shear thinning high molecular weight β-glucan based polysaccharide to xanthan gum for the management of patients with oropharyngeal dysphagia. It is therefore important to understand more fully its hydrodynamic properties in solution, in particular heterogeneity, molecular weight distribution and its behaviour in the presence of mucin glycoproteins. A commercially purified scleroglucan preparation produced by fermentation of the filamentous fungus *Sclerotium rolfsii* was analysed in deionised distilled water with 0.02% added azide. Sedimentation velocity in the analytical ultracentrifuge showed the scleroglucan preparation to be unimodal at concentrations >0.75 mg/ml which resolved into two components at lower concentration and with partial reversibility between the components. Sedimentation coefficient versus concentration plots showed significant hydrodynamic non-ideality. Self-association behaviour was confirmed by sedimentation equilibrium experiments with molecular weights between ~3 × 10^6^ g/mol to ~5 × 10^6^ g/mol after correcting for thermodynamic non-ideality. SEC-MALS-viscosity experiments showed a transition between a rod-shape at lower molar masses to a more flexible structure at higher masses consistent with previous observations. Sedimentation velocity experiments also showed no evidence for potentially problematic interactions with submaxillary mucin.

## Highlights

• Scleroglucan considered as alternative to xanthan.
• Sedimentation velocity shows partial reversible self-association.
• Associative behaviour confirmed by sedimentation equilibrium.
• SEC-MALS and viscosity show transition between a rod to more flexible structure.
• No problematic interactions with submaxillary mucin.

## Graphical abstract

**Figure:**
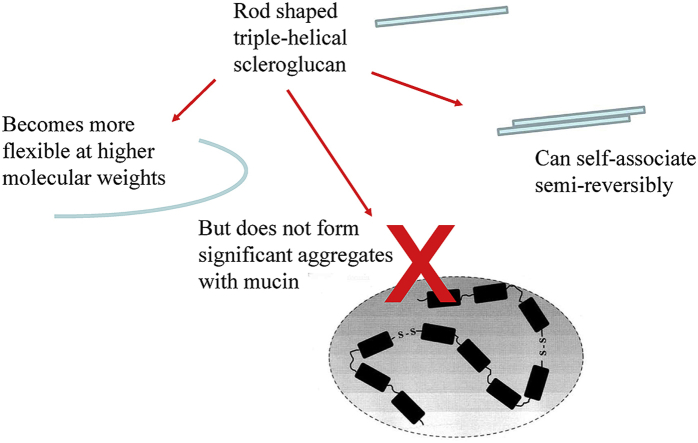

## 1 Introduction

Oropharyngeal dysphagia is known as swallowing disorder in the upper aero digestive tract (Martino, Pron, & Diamant, 2000). The first polysaccharide to be considered for the management of dysphagia was starch but the pasty turbid products arising from poor solubility proved unpopular with patients. Xanthan gum – which gives clearer solutions – is now being considered and there has been no evidence of any residue left in the oral cavity after the use of xanthan gum based thickeners on dysphagia patients (Rofes, Arreola, Mukherjee, Swanson, & Clavé, 2014).

Xanthan is a large molecular weight (~3 × 10^6^ g/mol) microbial/fungal polysaccharide which gives high viscosities and provides strong thickening to liquid based foods. The primary structure of xanthan gum consists of the cellulose-like backbone of (1→4)-linked β-d-*Glc*p residues substituted at O-3 of alternate glucose residues, with a trisaccharide side chain. The trisaccharide side chain consists of a β-d-*Man*p-(1→4)-β-d-*Glc*pA-(1→2)-α-d-*Man*p- unit. Non-carbohydrate substituents include an acetyl group at O-6 of the inner *Man*p residue and a pyruvate group at O-4,6 of the terminal *Man*p. The pyruvic acid content of xanthan can vary according to the producing bacterial strain. It is a dimeric helical polysaccharide which adopts an extended conformation in solution (Berth et al., 1996) with a large persistence length (a measure of chain rigidity/flexibility of polysaccharides). The extended rod-like characteristic renders it susceptible to shear thinning in solution, a property which is desirable for managing dysphagia. Scleroglucan is another microbial/fungal large molecular weight β-glucan based polysaccharide, produced from the filamentous fungus *Sclerotium rolfsii*. It is being considered as an alternative rod-like, shear thinning high molecular weight β-glucan based polysaccharide. The possible uses of scleroglucan across a wide range of sectors have already been well described (see, for example, Harding et al., 2017; Morris & Harding, 2009). It has been considered for example for use in cosmetics (as part of skin and hair products), for application in pesticides (to assist binding to foliage), and, along with xanthan and other polysaccharides, as a water immobiliser and hydrogel (Survase et al, 2007) in drug delivery. A further aspect of water immobilization ability has been taken advantage of for binding water and providing high heat stability in oil well drilling fluids. In common with schizophyllan – another β(1 → 3)-linked glucan with β(1 → 6)-linked branches – an earlier study has suggested it may stimulate an immune response against tumour cells (Kurachi, Ohno, & Yadomae, 1990).

**Fig. 1:**
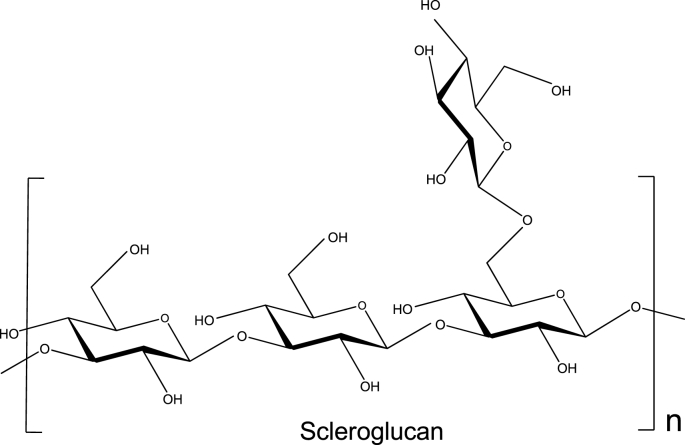
Primary structure of scleroglucan (Courtesy of Dr. C. Lawson, Carbosynth, Compton UK).

Key to these possibilities has been the size, conformation and conformational rigidity of the scleroglucan molecule which has made it distinct from more flexible classes of polysaccharide. Scleroglucan (Fig. 1) is a beta-glucan whose primary structure is a backbone of repeating β(1 → 3)-linked glucose residues:

… →3) β-d-Glc*p*-(1→ …

and approximately every third residue has a β(1 → 6)-linked d-glucose branch (Rinaudo & Vincendon, 1982; Farina, Sineriz, Molina, & Perotti, 1998). Previous studies largely based on light scattering (Lecacheux, Mustiere, Panaras, & Brigand, 1986; Yanaki et al., 1983, 1981) have suggested scleroglucan preparations have average molecular weights up to 6 × 10^6^ g/mol, with considerable polydispersity. X-ray fibre diffraction studies have suggested that in an ordered form they exist as hydrogen-bond stabilized triple helices (Gawronski & Park, 1999) with the β(1 → 6)-linked d-glucose branches protruding from the triple helical axis of the molecule. The existence of a trimeric structure has been supported by light scattering studies, by comparison of the molecular weights in non-dissociative and dissociative solvents for two different preparations – one sonicated, one not – of different molecular weight (Yanaki, Kojima, & Norisuye, 1981, 1983).

A consequence of the oligomeric helical structure is, in common with schizophyllan, an extended conformation in solution with a large persistence length (average projection from one end of the molecule to the original direction of the other end, in the limit the chain length → ∞) namely ~ 200 nm (Biver, Lesec, Allain, Salome, & Lecourtier, 1986). A consequence of the extended characteristics of the scleroglucan molecule is the shear thinning property of more concentrated solutions similar to xanthan which has also an extended conformation with high persistence lengths (Berth et al., 1996). However earlier papers have reported further associative effects beyond the “trimer” (Lecacheux et al., 1986; Yanaki, 1983).

It is therefore important to understand more fully its hydrodynamic properties in solution, in particular heterogeneity, molecular weight distribution and particularly its behaviour in the presence of mucin glycoproteins to check for the possibility of potentially problematic aggregation interactions, which could cause problems for dysphagia patients. We do this by taking advantage of recent advances in both sedimentation velocity and sedimentation equilibrium in the analytical ultracentrifuge (AUC), together with advances in size-exclusion chromatography coupled to multi-angle light scattering and differential pressure viscometry.

## 2 Materials and methods

### 2.1 Materials

Scleroglucan extracted from *Sclerotium rolfsii* was supplied from Carbosynth Ltd. (Compton, UK). It was suspended in triply distilled deionised water, and further purified by centrifugation and dialysis. The 0.2% (w/v) solution was centrifuged at 4850 g and 20.0 °C for 30 min to remove any supramolecular species. The supernatant was then dialyzed in water to remove any low molecular weight (<14 kDa maximum) impurities. Then the dialyzed supernatant was freeze dried in an Edwards Moduylo (York, UK) freeze drier. An appropriate amount of the freeze dried scleroglucan sample was resuspended in a 0.02% sodium azide solution to prepare a 1% scleroglucan stock solution. Suspension in additional low molecular weight electrolyte to suppress any polyelectrolyte behaviour (as needed for example for the polyanionic xanthan) was unnecessary, as, unlike xanthan, scleroglucan is a neutral, uncharged polysaccharide. To prepare more diluted scleroglucan solutions (0.2% and 0.5%), the stock solution was diluted with the appropriate amount of aqueous solution of 0.02% (w/v) sodium azide and mixed on a magnetic stirrer at 20.0 °C for > 2 h.

For the interaction studies, human maxillary mucin was not available so bovine submaxillary mucin (Dinu et al., 2019) was used instead. For these experiments suspension in phosphate buffered saline, mimicking the inorganic conditions in the mouth, with pH 6.8, ionic strength I = 0.10 (and suppressing polyelectrolyte behaviour in the polyanionic mucin) was used (Sarkar, Goh, & Singh, 2009).

### 2.2 Sedimentation velocity in the analytical ultracentrifuge

Sedimentation coefficient distributions were evaluated using the Beckman Optima XL-I analytical ultracentrifuge (Beckman Instruments, Palo Alto, USA). A volume of 400 μl of scleroglucan solution and matching amounts of solvent (water) were injected into appropriate channels of 12 mm double sector aluminium epoxy cells with sapphire windows. A range of concentrations from 0.075 to 2.0 mg/ml were studied. Solutions were centrifuged at 30000–45000 rpm at a temperature of (20.0 ± 0.1) °C. The data was analysed by using the least squares g(*s*) model in SEDFIT (Dam & Schuck, 2004). The weight average sedimentation coefficient ‘*s*’ (in Svedbergs, S, with 1S = 10^−13^ s) for a particular component was then corrected to standard solvent conditions (the density and viscosity of water at a temperature of 20.0 °C), *s*_20,w_ (Van Holde, 1971, pp. 171–172). A polyglucose partial specific volume $\bar{v}$ of 0.61 ml/g was used (Determan, 2012).

### 2.3 Sedimentation equilibrium in the analytical ultracentrifuge

Sedimentation equilibrium experiments were also performed on the Optima XL-I. 20 mm long optical path length double-sector cells (Nanolytics, Potsdam, Germany) with sapphire windows were loaded with 80 μl of dialysed sample and a matching amount of reference buffer dialysate in appropriate channels. The balanced cells were then loaded into an analytical 8-hole titanium rotor An50-Ti and placed in the AUC. After time was allowed for vacuum formation and for temperature equilibration (20.0 °C), the rotor was accelerated to 3000 rpm. Using the Rayleigh interference optical system, scans were taken every 1 h and equilibrium was reached after approximately 72 h. Records of the relative concentration distribution of the solute at equilibrium was analysed to give the (whole distribution) apparent weight average molecular weight *M*_w,app_ using the SEDFIT-MSTAR algorithm (Schuck et al., 2014). It uses the M* function of Creeth and Harding (1982) particularly suited for the analysis of difficult heterogeneous systems, and a second method known as the hinge point used as an internal check on the result. Point (apparent) weight average molecular weights *M*_w,app_(*r*) were also estimated from local slopes of the ln*c*(*r*) vs *r*^*2*^ plots. Scleroglucan solutions are known to be thermodynamically non-ideal, even in dilute solution (Harding, 1992; Lecacheux et al., 1986), and correction from apparent molecular weights to ‘ideal’ molecular weights *M*_w_ was made using the relation (1) (Tanford, 1961) assumed valid in dilute solution (<0.5 mg/ml).

(1) $$M_{w}=M_{w,app}\cdot \left(1+2BM_{w}c\right)$$

### 2.4 Capillary viscometry

Intrinsic viscosities were measured using a standard Schott-Geräte Ostwald 2 ml capillary viscometer, with automatic timing and water bath equilibrated to (20.00 ± 0.01) ^o^C. A series of concentrations of scleroglucan solutions were injected to the reservoir of the capillary tube. Then the solutions were pumped to the upper line of the capillary. The time was measured when solution fell from the upper line to the bottom line of the capillary.

Relative specific viscosities η_r_ (the ratio of the solution viscosity to the solvent viscosity) were measured from the ratio of flow times of solution and solvent (because of high dilutions, c < 0.1 mg/ml, no density correction was necessary (see, e.g., Harding, 1997)) and reduced viscosities η_red_ obtained from:

(2) $$\eta_{red}=\frac{\eta_{r}-1}{c}$$

The intrinsic viscosity [η] – the limiting value of the reduced viscosity at *c* = 0 was then estimated using three different extrapolaton methods. Firstly the standard Huggins (1942) extrapolation:

(3) $$\eta_{red}=\left[\eta \right]\cdot \left(1+K_{H}\left[\eta \right]c\right)$$

*K*_*H*_ is the Huggins coefficient. Secondly the Kraemer (1938) extrapolation of the inherent viscosity

$$\eta_{inh}=\ln\{\eta_{r}/c\}$$

(4) $$\eta_{inh}=\left[\eta \right]\cdot \left(1-K_{K}\left[\eta \right]c\right)$$

where *K*_*K*_ is the Kraemer coefficient, was used and finally the intrinsic viscosity was estimated at each concentration using the Solomon-Ciuta equation (1962):

(5) $${\left[\eta \right]}_{SC}\sim \left(1/c\right)\cdot {\left[2\{\eta_{sp}-\ln(\eta_{r})\}\right]}^{1/2}$$

### 2.5 Size exclusion chromatography coupled to multi-angle light scattering (SEC-MALS) and differential pressure viscometer

A Schimadzu LC-20 HPLC system (Shimadzu Europe, Duisburg, Germany) comprising a DGU-20A degassing unit, LC-20AD solvent delivery system and SIL-20AHT autosampler, delivered 0.1M sodium nitrate/0.02% azide at 0.5mL/min to a Tosoh PWXL guard column. The guard column was serially connected to two Tosoh TSK-gel size-exclusion columns (G6000 PWXL followed by G5000 PWXL). Light scattering intensity was detected using a DAWN® HELEOS™ 18 angle light scattering photometer connected in series to an ViscoStar® II on-line differential pressure viscometer, Optilab® T-REX refractive index detector (Wyatt Technology Corporation, California, U.S.A.). The stock solution of 5.0mg/ml was filtered through a 0.45 μm syringe filter (Whatman, Maidstone, England) - to remove any insoluble material or dust prior to injection - and then diluted to 1 and 2.5mg/ml. A 100μL aliquot of each solution was without further filtering injected onto the columns at ambient temperature. ASTRA™ (Version 6) software (Wyatt Technology Corporation, California, U.S.A.) was used to estimate the weight average *M*_w_(*V*_e_) and relative viscosities η_r_(*V*_e_) as a function of elution volume *V*_e_. A refractive increment (*dn/dc*) ~ 0.14 mL/g (Lecacheux et al., 1986) was used and because of the high dilution the second virial coefficient *B* was set to zero.

The differential pressure drop from solution flow versus solvent flow yielded η_r_ and then the reduced viscosity η_red_(*V*_*e*_) at each elution volume *V*_*e*_ from this form of eq. (2):

(2b) $$\eta_{red}\left(V_{e}\right)=\{\eta_{r}\left(V_{e}\right)-1\}/c\left(V_{e}\right)$$

Because no Huggins or Kraemer extrapolation to zero concentration is possible, intrinsic viscosities at each elution volume [η](*V*_e_) were obtained using the Solomon-Ciuta equation as a function of elution volume *V*_e_, in this form of eq. (5):

(5b) $$\left[\eta \right]\left(V_{e}\right)\sim \left[1/c\left(V_{e}\right)\right]\cdot {\left[2\{\eta_{sp}\left(V_{e}\right)-\ln\;\eta_{r}(V_{e})\}\right]}^{1/2}$$

## 3 Results and discussion

### 3.1 Heterogeneity: sedimentation coefficient and sedimentation coefficient distribution

**Fig. 2:**
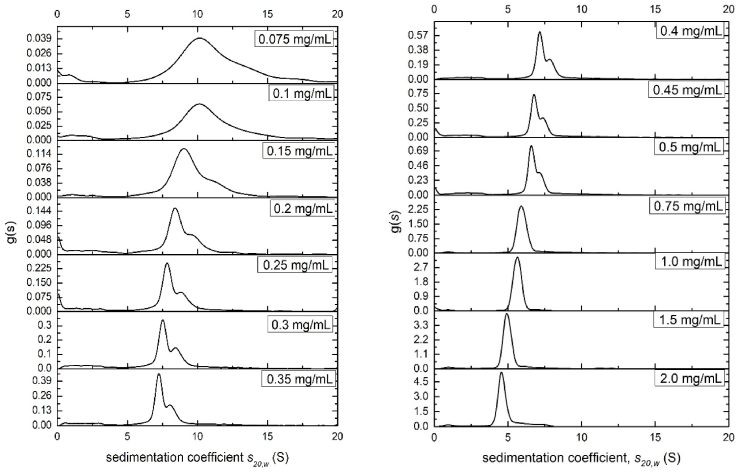
Sedimentation coefficient distributions g(*s*) vs *s*_20,w_ for different loading concentrations of scleroglucan in deionised distilled water supplemented with 0.02% azide. Of the two main components on average (65.5 ± 2.5) % is the slower component and (34.5 ± 2.5) % is the faster component.

**Fig. 3:**
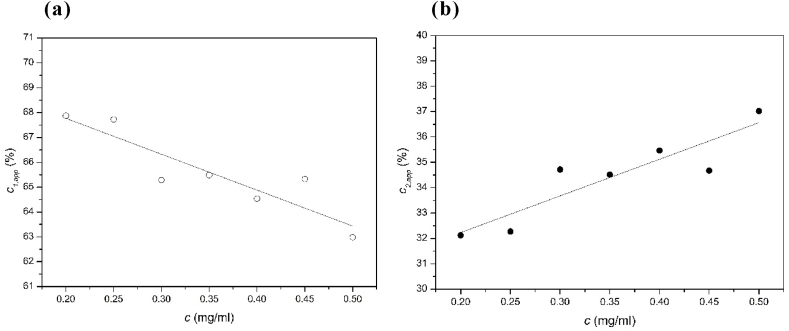
Plot of the relative concentration of (a) the 1st main component, c_1_, as a function of loading concentration c, and (b) the 2nd main component, c_2_, as a function of c.

Sedimentation velocity in the analytical ultracentrifuge showed the scleroglucan preparation was unimodal at concentrations >0.75 mg/ml which resolved into two main components at lower concentration and with partial reversibility between the components (Fig. 2), accounting for ~ 80% of the total distribution. What is interesting is the relative proportion of these two main components changes: increasing the loading concentration results in an increase in the faster or higher molecular weight component compared with the slower component. Fig. 3 shows this change: across a concentration range from 0.2 to 0.5 mg/ml results in a 5% drop of the lower molecular weight/slower sedimenting component and a corresponding increase in the higher molecular weight/faster sedimenting component was found. This behaviour is commensurate with a partially reversible association-dissociation. This trend is opposite to what might be expected from classical Johnston-Ogston effects (Johnston & Ogston, 1946) which can result in a diminution of the apparent concentration *c*_2_ of the faster moving component with increase in total loading concentration *c* (=*c*_1_+*c*_2_). The fast component is impacted more by the hydrodynamic non-ideality. With the increase of the concentration, the fast component “2” is slowed down because it moves through the main slower component “1”. By contrast, the slow component is not slowed down as much as the fast component because it only needs to move through the solvent, which is less viscous. Therefore, there appears only one peak at higher concentrations where the slower component catches up the faster component. However, when the concentration is too low, there was not enough sensitivity for the AUC to catch the signal of two peaks. Therefore, there is again only one clear peak when the concentration is lower than 0.1 mg/ml.

**Fig. 4:**
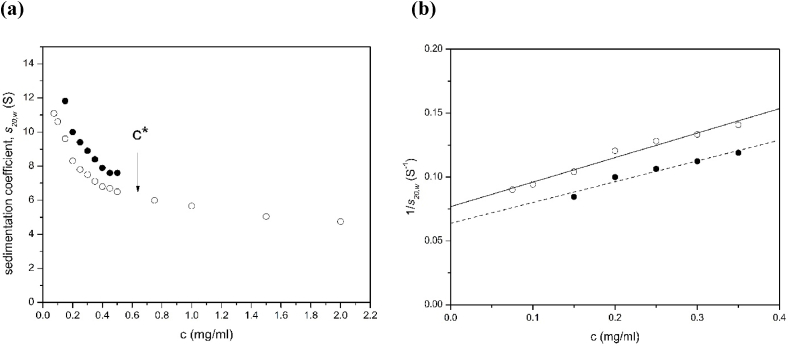
Plot of (apparent) sedimentation coefficient of sclerogucan in aqueous solution (water as solvent, supplemented with 0.02% azide) for the slower component (open circles) and faster component (solid circles) corrected to standard solvent conditions, i.e. the viscosity, density of water at 20.0 °C (b) extrapolation of the reciprocal of the sedimentation coefficient *s*_20,w_ to zero concentration to yield *s*^o^_20,w_. Values of *s*^o^_20,w_ = (13.0 ± 0.5)S and *s*^o^_20,w_ = (15.7 ± 1.3)S for the slow and fast components respectively were obtained.

The sedimentation coefficient versus concentration plots for both components showed significant hydrodynamic non-ideality (Fig. 4), due to exclusion volume and viscosity effects. In Fig. 4, *s* values had been corrected to standard conditions (density and viscosity of water at 20.0 °C). There appeared to be a discontinuity between 0.4 and 0.75mg/ml after which the second component has not resolved from the main peak. This could correspond to (i) the dilute solution limit or c* (Cuvelier & Launay, 1986): a similar value was seen for xanthan (Dhami et al., 1995); (ii) increased hydrodynamic non-ideality retarding more the faster component which is moving through a solution of the slower component. Linear extrapolation of datasets of 1/*s*_20,w_ vs total concentration, *c*, to *c* = 0 for values of *c* < 0.4 mg/ml yielded estimates for the main (slower) component of *s*^o^_20,w_ = (13.0 ± 0.5)S and for the faster component of *s*^o^_20,w_ = (15.7 ± 1.3)S (Table 1).

**Table 1:** Hydrodynamic properties of scleroglucan in aqueous solution (20.0 °C, water solvent).

Parameter	
*s*^o^_20,w_ (component 1)	(13.0 ± 0.5) S
% (component 1)^a^	65.5 ± 2.5
*s*^o^_20,w_ (component 2)	(15.7 ± 1.3) S
% (component 2)^b^	34.5 ± 2.5
*M*_w_ (whole distribution weight average)	(2.8 ± 0.3) x 10^6^ g/mol
[η]	(2190 ± 110) ml/g
MHKS *a* (lower molecular weights)	1.14 ± 0.03
MHKS *a* (higher molecular weights)	0.76 ± 0.01

a Mean value – decreases with increase in total concentration.

b Mean value – increases with increase in total concentration.

### 3.2 Partially reversible self-association

**Fig. 5:**
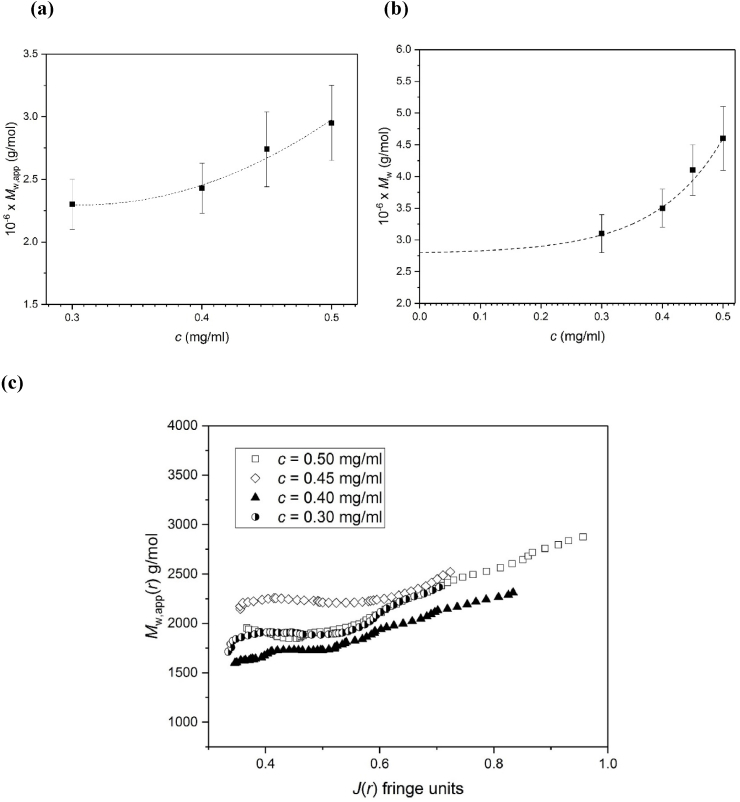
Sedimentation equilibrium of scleroglucan. (a) Plot of (whole distribution) apparent weight average molecular weight *M*_w,app_ versus cell loading concentration *c* scleroglucan in deionised distilled water supplemented with 0.02% azide.; (b) after correction for non-ideality, plot of (ideal) *M*_w_ versus *c*; (c) plot of point or local average molecular weight *M*_w,app_(*r*) as a function of local (Rayleigh fringe) concentrations *J*(*r*) at radial positions *r* for different cell loading concentrations.

The partially reversible self-association inferred from the sedimentation velocity results appear to be consistent with sedimentation equilibrium, with an increase in the (whole distribution) apparent weight average molecular weight *M*_w,app_ as a function of loading concentration *c* (Fig. 5a). This increase overcomes the effects of thermodynamic non-ideality which would normally lead to a decrease in *M*_w,app_ with increase in concentration, *c*. This tendancy to self-associate, also reported by Lecacheux et al. (1986) , on the basis of low-angle light scattering measurements is not seen in xanthan which follows the classical decrease with *c* (Dhami et al., 1995): in such cases extrapolation to *c* = 0 effectively negates these effects to yield a true ideal weight average molecular weight. It is possible to compensate for these effects for a self-associating system if we have an estimate for the non-ideal contribution to *M*_w,app_. In this regard, in the dilute solution region the relation given in equation (1), namely (1/*M*_w,app_) = (1/*M*_w_) (1 + 2*BM*_w_*c*) applies where *B* is the second thermodynamic virial coefficient. The factor (1 + 2*BM*_w_*c*) represents the factor by which *M*_w,app_ underestimates the true or ideal weight average molecular weight *M*_w_, at a concentration *c*. For scleroglucan *BM*_w_ ~570 ml/g (Harding, 1992; Lecacheux et al., 1986) and it is possible to convert the plot of *M*_w,app_ vs *c* (Fig. 4a) to *M*_w_ vs *c* (Fig. 5b) further emphasizing the effect of self-association. From extrapolation we obtain an estimate for *M*_w_^o^, the value of *M*_w_ in the zero concentration limit, of ~ (2.8 ± 0.3) x 10^6^ g/mol, comparable with that for xanthan (Dhami et al., 1995). Up to the apparent dilute solution limit, *M*_w_ rises to ~4.5 × 10^6^ g/mol.

To examine the reversibility of the association we used the diagnostic technique of overlap of data-sets of point average apparent molecular weight *M*_w,app_(*r*) vs local (fringe) concentration *J*(*r*) as a function of radial position *r* in an ultracentrifuge cell for different cell loading concentrations *c* (Creeth & Harding, 1982b; Nikolajski et al., 2014; Roark & Yphantis, 1969). For a completely reversible association-dissociation the different data-sets obtained at different loading concentrations overlap and form part of the same curve. Compared to aminocellulose for example (Nikolajski et al., 2014) only partial overlap is seen (Fig. 5c) indicating partial reversibility.

### 3.3 Intrinsic viscosity

**Fig. 6:**
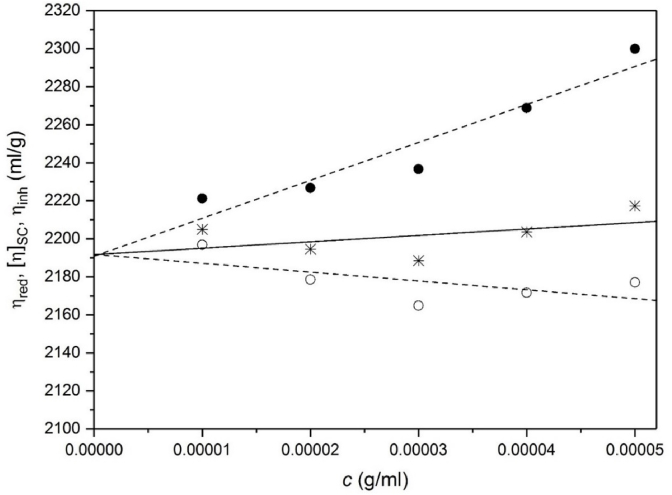
Intrinsic viscosity estimations from the Ostwald viscometer for scleroglucan in deionised distilled water supplemented with 0.02% azide. Filled circles – reduced viscosities η_red_, yielding a value of [η] or (2191 ± 110) ml/g. Open circles – inherent viscosities η_inh_, yielding a value of [η] or (2192 ± 110) ml/g. Stars – Solomon-Ciuta values [η]_SC_ yielding a value of [η] or (2192 ± 110) ml/g.

Fig. 6 shows the different extrapolations to estimate the intrinsic viscosity [η] using the Ostwald viscometer. The assumption is made, as before with xanthan (Dhami et al., 1995), that due to the slow creeping flow conditions non-Newtonian flow effects are not significant. Flow time increments between solution and solvent were very large so very low concentrations could be employed, <0.05 mg/ml and well below the apparent c*. Both the extrapolation methods – Huggins and Kraemer – gave very similar values for the intrinsic viscosity, (2191±110)ml/g and (2192±110)ml/g respectively. Within this concentration range the Solomon-Ciuta estimates for [η] at each concentration were reasonable and unsurprisingly, since it is based on a combination of the two other extrapolation methods gave an extrapolated value to *c* = 0 in agreement with the other two. The intrinsic viscosity is lower than the intrinsic viscosity of xanthan gum measured in previous studies (Dhami et al., 1995).

### 3.4 Conformation

**Fig. 7:**
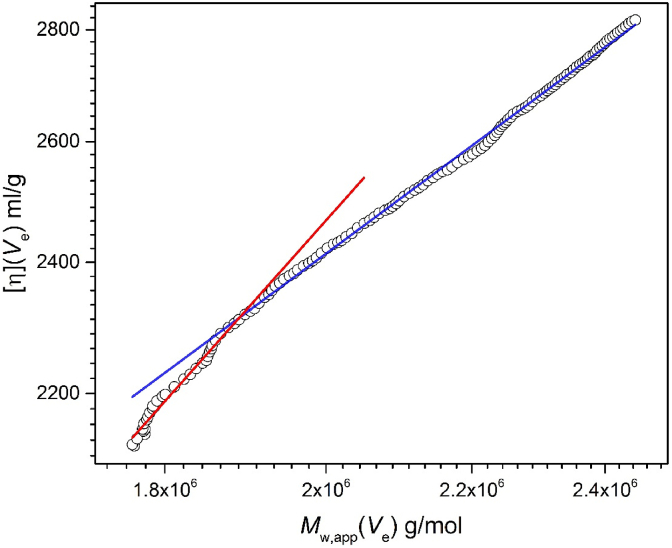
Mark-Houwink-Kuhn-Sakurada plot of intrinsic viscosity [η](*V*_e_) and weight average molecular weight *M*_w_(*V*_e_)as a function of elution volume *V*_e_. The initial limiting slope (red) = (1.14 ± 0.03), corresponding to an extended rod-like structure. The final limiting slope at high molecular weight (blue) = (0.76 ± 0.01), corresponding to a more flexible structure. (For interpretation of the references to colour in this figure legend, the reader is referred to the Web version of this article.)

We use size exclusion chromatography coupled to multi-angle laser light scattering (SEC-MALS) to give an indication of the conformation/chain rigidity of the scleroglucan. Although SEC-MALS is not the first method of choice for studying very large polymers due to the limit of resolution of the SEC columns, when coupled to an on-line differential pressure viscometer (and using the approximate Solomon-Ciuta equation to estimate [η]) equation it is still possible to estimate the conformation from the dependence of the intrinsic viscosity [η](*V*_e_) relationship with *M*_w,app_(*V*_e_) across a range of elution volumes *V*_e_ using for example the Mark-Houwink-Kuhn-Sakurada (MHKS) power law relation (see for example Harding, Vårum, Stokke, & Smidsrød, 1991;Smidsrød & Andresen, 1979; Tsvetkov, Eskin, & Frenkel, 1970):

(6) $$\left[\eta \right]\;=\;\kappa_{\eta }{M_{w}}^{a}$$

where κ_η_ and *a* are characteristic coefficients related to conformation. Fig. 7 shows the double-logarithmic MHKS plot of [η](*V*_e_) versus *M*_w_(*V*_e_): there appears a change in slope from a stiffish rod shape molecule (slope or “*a*” value of 1.14 ± 0.03) to a lower value as the molecular weight increases (*a* = 0.76 ± 0.01), corresponding to a more flexible structure. We were limited to the molecular weight range we could look at due to the finite separation range of the SEC columns for high molecular weight material but nonetheless the trend is visible. This is reasonably similar to what was observed by Yanaki et al. (1981) although they found even stiffer rods at lower molecular weights but those researchers did not have the benefit of on-line separation/viscosity/molecular weight instrumentation and their conclusions were limited to just two data points. We also observe considerable chain flexibility at higher molecular weights.

These observations appear to further reinforce the earlier observations of Lechacheux et al. (1986) and Yanaki et al. (1981, 1983) of a stiff conformation at lower molecular weights or chain lengths, becoming more flexible at higher molecular weights. We observe that the lower molecular weight structures (*M* ~<2 × 10^6^ g/mol) are stiff structures with xanthan-like properties (Berth et al., 1996) but above that they become considerably more flexible.

### 3.5 Molecular weight distribution and comparison with xanthan

As a final comparison with xanthan we can use the *Extended Fujita* method (Harding et al., 2011) to transform the sedimentation coefficient distribution g(*s*) at low concentration (to minimise non-ideality effects) to a molecular weight distribution f(*M*) vs *M*. The approach uses the power-law or scaling relationship between the sedimentation coefficient and molecular weight, analogous to the MHKS equation for viscosity (Eq. (6)) above (Harding et al., 1991; Smidsrød & Andresen, 1979; Tsvetkov et al., 1970):

(7a) *s = *κ_*s*_*M*_*w*_^*b*^

where κ_s_ and *b* are characteristic coefficients related to conformation. For example, *b* = 0.4–0.5 for a coil type of conformation, ~0.15–0.2 for a rod conformation and ~0.67 for a spherical conformation. If κ_s_ and *b* are known then *M*_w_ can be found from:

(7b) $$M_{w}={\left(\frac{s}{\kappa_{s}}\right)}^{1/b}$$

Fujita (1962) provided the basis for converting a (differential) distribution g(*s*) of the sedimentation coefficient *s* into a (differential) distribution f(*M*) of the molecular weight *M* for linear polymers, based on the assumption that the polymers behave as randomly coiled polymers in solution, with *b* = 0.5 in Eq. (7). The distribution function is

(8) f(M)=g(s)⋅(ds/dM)

where

(9) $$ds/dM=b\cdot \kappa_{s}^{1/b}\cdot s^{\left(b-1\right)/b}$$

Therefore, to perform the transformation the conformation type, *b* needs to be known under the particular solvent conditions and at least one pair of *s-M* values is needed to define the κ_s_, and this is what we have now applied to the xanthan and scleroglucan.

**Fig. 8:**
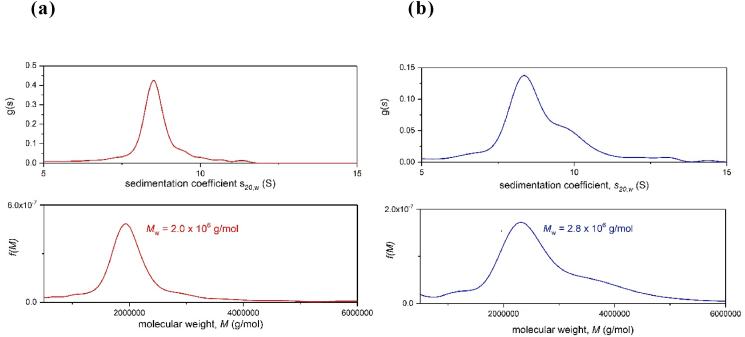
Transformation of sedimentation coefficient distributions g(*s*) vs *s* (for a loading concentration of 0.2 mg/ml) to molecular weight distributions f(*M*) vs *M*. (a) Xanthan (b) scleroglucan. Distributions – and weight averages - are similar.

*Defining b and* κ_*s*_: we use the Tsvetkov et al. (1970) relation linking *b* with *a*:

(10) $$b=\frac{2-a}{3}$$

since *a* = 1.23 for xanthan (Morris & Harding, 2013), *b* = 0.26. For scleroglucan, from the (mean) MHKS *a* value of 0.95, this leads to *b* = 0.35 across the distribution. To obtain κ_s_ we simply combine *b* with the “ideal” weight average molecular weight (Table 1) with the (weight average) sedimentation coefficient *s*_20,w_, at the concentration used for the distribution, using equation (7). A loading concentration of 0.2 mg/ml was used to minimize non-ideality effects. This yields values of 0.197 for xanthan (Morris & Harding, 2013) and 0.0492 for scleroglucan, respectively. The corresponding transformations from g(*s*) vs *s* to f(*M*) vs *M* are given in Fig. 8a for xanthan and Fig. 8b for scleroglucan.

In Eq. (7b) the weight average sedimentation coefficient s_20,w_ for the distribution (at 0.2 mg/ml) is combined with the (*ideal or zero concentration*) weight average molecular weight. In terms of molecular weight distribution it can be seen that both distributions are very similar, although scleroglucan has a more marked 2nd component of higher molecular weight as we have previously observed.

### 3.6 Sedimentation of mixtures of scleroglucan with bovine submaxillary mucin

**Fig. 9:**
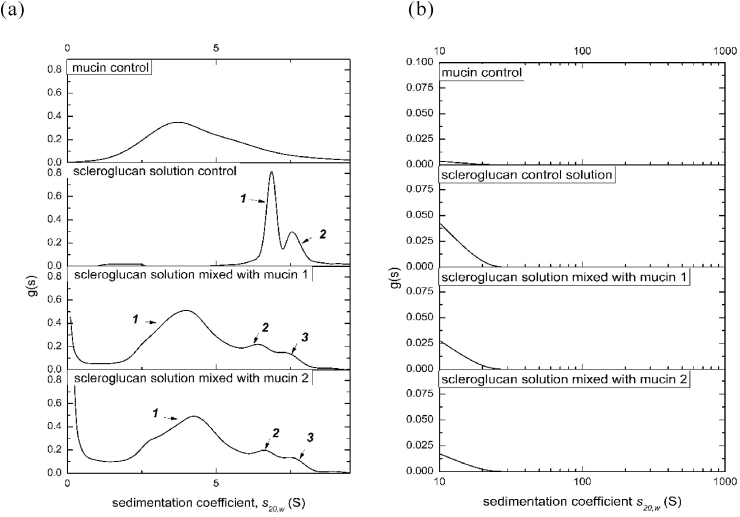
Sedimentation coefficient distributions g(*s*) vs *s*_20,w_ for mixtures of scleroglucan with bovine submaxillary mucin (BSM). (a) Sedimentation coefficient range: 0.1 to 10S, (b) 10 to 1000S. Final loading concentrations: scleroglucan 0.42 mg/ml, BSM 0.50 mg/ml.

We explored, using analytical ultracentrifugation, the behaviour of mixtures of scleroglucan with submaxillary mucin (using bovine submaxillary mucin, BSM, as the model mucin system – Dinu et al., 2019) to assay for the possible presence of deleterious large aggregation effects which might diminish the potential of scleroglucan as a dysphagia agent (Rofes et al., 2014). Experiments were conducted in phosphate buffered saline, mimicking the inorganic conditions in the mouth (and suppressing polyelectrolyte behaviour in the polyanionic mucin). The BSM solution and each biopolymer solution were separately mixed with a weight ratio of 1:5. Fig. 9 shows the sedimentation coefficient distribution plots for scleroglucan/BSM mixtures compared to controls, from the range 0-10S (Figs. 9a) and 10-1000S (Fig. 9b). From Fig. 9a the reduction and slight retardation of the faster moving scleroglucan peaks on mixing may be due to Johnston-Ogston effects (faster components slowed down by the viscosity of the slower components – as considered above), but there is no evidence for faster moving large aggregates at least not in the size range up to 1000S (Fig. 9b).

## 4 Concluding remarks

Our study confirms previous work that scleroglucan is a large and complex microbial polysaccharide in aqueous solution, consistent with previous observations of a tendency to self-associate beyond the trimeric, triple helical structure observed by others, and in a semi-reversible manner. In some ways it has properties similar to xanthan, another microbial/fungal polysaccharide with a β(1→4) backbone and of a similar molecular weight – and molecular weight distribution, and which also exhibits complex properties. Encouragingly, in terms of its potential use for the treatment of dysphagia, scleroglucan showed no evidence from this study for significant interactions with submaxillary mucin, nor the formation of large mucoadhesive aggregates seen for other types of polysaccharide-mucin system & hence reduce the likelihood of choking. From the conformation work it seems that scleroglucans of molecular weight <1.9 × 10^6^ g/mol may be worthy of further consideration as an alternative to xanthan for the treatment/management of patients with oropharyngeal dysphagia (Martino et al., 2000).

## Declaration of competing interest

There are no conflicts of interest.
